# Quantitative susceptibility mapping reveals widespread brain iron abnormalities in sporadic patients with early-stage amyotrophic lateral sclerosis

**DOI:** 10.1093/braincomms/fcag190

**Published:** 2026-05-26

**Authors:** Mingjie Ma, Bing Zhao, Ninglu Gao, Xiaohan Sun, Kai Shao, Pengfei Lin, Wei Li, Yuying Zhao, Dexin Yu, Chuanzhu Yan, Shuangwu Liu, Yan Yun

**Affiliations:** School of Nursing and Rehabilitation, Cheeloo College of Medicine, Shandong University, Jinan 250012, China; Department of Neurology, Research Institute of Neuromuscular and Neurodegenerative Diseases, Shandong Provincial Key Laboratory of Mitochondrial Medicine and Rare Diseases, Qilu Hospital, Cheeloo College of Medicine, Shandong University, Jinan 250012, China; Department of Neurology, Qilu Hospital (Qingdao), Cheeloo College of Medicine, Shandong University, Qingdao 266035, China; Department of Neurology, Research Institute of Neuromuscular and Neurodegenerative Diseases, Shandong Provincial Key Laboratory of Mitochondrial Medicine and Rare Diseases, Qilu Hospital, Cheeloo College of Medicine, Shandong University, Jinan 250012, China; Department of Neurology, Research Institute of Neuromuscular and Neurodegenerative Diseases, Shandong Provincial Key Laboratory of Mitochondrial Medicine and Rare Diseases, Qilu Hospital, Cheeloo College of Medicine, Shandong University, Jinan 250012, China; Department of Clinical Laboratory, Qilu Hospital (Qingdao), Cheeloo College of Medicine, Shandong University, Qingdao 266035, China; Department of Neurology, Research Institute of Neuromuscular and Neurodegenerative Diseases, Shandong Provincial Key Laboratory of Mitochondrial Medicine and Rare Diseases, Qilu Hospital, Cheeloo College of Medicine, Shandong University, Jinan 250012, China; Department of Neurology, Research Institute of Neuromuscular and Neurodegenerative Diseases, Shandong Provincial Key Laboratory of Mitochondrial Medicine and Rare Diseases, Qilu Hospital, Cheeloo College of Medicine, Shandong University, Jinan 250012, China; Department of Neurology, Research Institute of Neuromuscular and Neurodegenerative Diseases, Shandong Provincial Key Laboratory of Mitochondrial Medicine and Rare Diseases, Qilu Hospital, Cheeloo College of Medicine, Shandong University, Jinan 250012, China; Department of Radiology, Qilu Hospital, Cheeloo College of Medicine, Shandong University, Jinan 250012, China; Department of Neurology, Research Institute of Neuromuscular and Neurodegenerative Diseases, Shandong Provincial Key Laboratory of Mitochondrial Medicine and Rare Diseases, Qilu Hospital, Cheeloo College of Medicine, Shandong University, Jinan 250012, China; Department of Radiology, Qilu Hospital, Cheeloo College of Medicine, Shandong University, Jinan 250012, China; School of Nursing and Rehabilitation, Cheeloo College of Medicine, Shandong University, Jinan 250012, China; Department of Neurology, Research Institute of Neuromuscular and Neurodegenerative Diseases, Shandong Provincial Key Laboratory of Mitochondrial Medicine and Rare Diseases, Qilu Hospital, Cheeloo College of Medicine, Shandong University, Jinan 250012, China; Department of Radiology, Qilu Hospital, Cheeloo College of Medicine, Shandong University, Jinan 250012, China

**Keywords:** ALS, quantitative susceptibility mapping, motor disability

## Abstract

In the present study, using the novel quantitative susceptibility mapping technique, we aimed to systematically investigate brain iron alterations in a large group of sporadic early-stage amyotrophic lateral sclerosis patients and their correlation with clinical disability. In this study, amyotrophic lateral sclerosis patients at King's stage 1 were defined as early-stage amyotrophic lateral sclerosis patients, and 53 newly diagnosed early-stage amyotrophic lateral sclerosis patients and 50 healthy controls were included. Voxel-based whole-brain quantitative susceptibility mapping analysis was used to explore brain iron alterations. Voxel-based morphometry analysis was also performed. Longitudinal follow-up was performed in amyotrophic lateral sclerosis patients, and the follow-up progression rate was calculated. We found that, compared with healthy controls, early-stage amyotrophic lateral sclerosis patients presented significantly increased susceptibility values, mainly in the motor cortex, prefrontal cortex, hippocampus and cerebellar regions, while volumetric alterations were not detected. Moreover, motor and extra-motor cortex susceptibility values were significantly correlated with upper motor neuron scores and follow-up progression rate (*r* = 0.452–0.504, *P* < 0.01) in early-stage amyotrophic lateral sclerosis patients. We demonstrated a clear profile of early motor and extra-motor iron depositions and their important roles in early-stage amyotrophic lateral sclerosis patients. We suggest that quantitative susceptibility mapping is likely a promising neuroimaging approach for assessing early upper motor neuron damage and detecting early extra-motor alterations in amyotrophic lateral sclerosis patients.

## Teaser

Ma *et al*. reported widespread brain iron accumulation in patients with early-stage amyotrophic lateral sclerosis, suggesting that quantitative susceptibility mapping may serve as a promising tool for detecting early motor and extra-motor involvement and for monitoring disease progression.

## Graphical Abstract

**Graphical Abstract:**
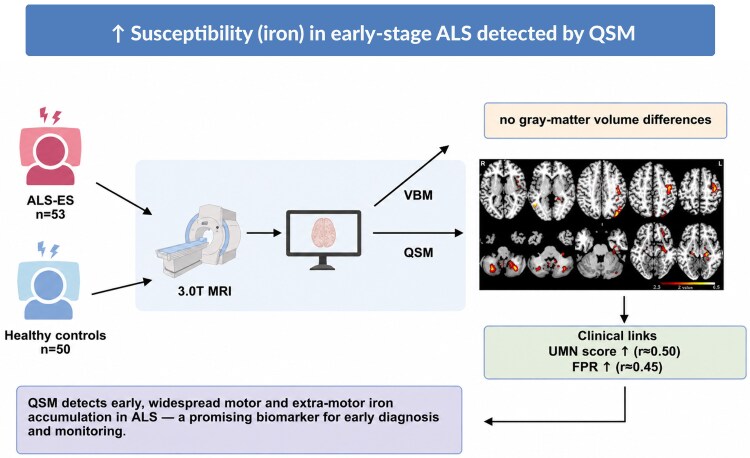

## Introduction

Amyotrophic lateral sclerosis (ALS) is currently considered a multisystemic disorder with widespread brain involvement.^1-3^ Increasing evidence indicates that iron metabolism abnormalities may play an important role in ALS pathology.^4,5^ Moreover, iron chelation treatments have been found to slow disease progression in ALS animals.^4,6^ Thus, early detection of abnormal whole-brain iron metabolism may provide a deeper *in vivo* understanding of neurodegeneration in ALS.^1-6^

Recently, using the novel quantitative susceptibility mapping (QSM) technique, an increasing number of neuroimaging studies have demonstrated that abnormal iron accumulation in the motor cortex is a consistent feature of ALS patients.^5,7-11^ Although these studies are insightful, several gaps remain. For example, most previous QSM studies have relied on ROI-based measurements centred on the motor cortex, which may introduce selection bias and overlook extramotor involvement, and many were performed in clinically heterogeneous cohorts at relatively advanced stages. Consequently, it remains unclear whether motor cortex iron deposition is already detectable in early-stage ALS and whether it is associated with motor disability, particularly UMN involvement.^5,7-11^ Moreover, longitudinal evidence linking baseline iron-related susceptibility changes to subsequent clinical progression is still limited.^5^ To address these gaps, we conducted voxel-wise whole-brain QSM in an early-stage sporadic ALS cohort (Gold Coast criteria; King stage 1) and performed a prospective follow-up to examine whether baseline susceptibility abnormalities are associated with subsequent ALSFRS-R decline.

To our knowledge, very few previous studies have used QSM to investigate whole-brain iron metabolism alterations in ALS, and these studies were conducted in relatively advanced-stage patients; thus, whether extramotor iron accumulation occurs in early-stage ALS remains unclear.^5,9-16^

Against this background, in this prospective study, we included a relatively large group of newly diagnosed sporadic early-stage ALS patients and healthy subjects. We had two aims. First, using voxel-based whole-brain QSM analyses, we aimed to identify a profile of whole-brain iron accumulation, including motor and extramotor regions, in sporadic patients with early-stage ALS. Based on the new Gold Coast criteria and previous studies, King's stage 1 ALS was defined as early-stage ALS (ALS-ES).^17,18^ Second, we aimed to explore the relationships between brain iron accumulation and clinical disabilities, particularly UMN damage and the follow-up progression rate (FPR), in early-stage ALS patients.

## Materials and methods

### Participants

In the present study, the inclusion criteria for ALS patients were as follows: (i) All patients met the Gold Coast criteria.^17^ (ii) ALS patients at King's stage 1 were defined as ALS-ES patients; thus, only ALS patients classified as King's stage 1 were included.^17^

The exclusion criteria for ALS patients were as follows: (i) Family history of ALS or carriers of known ALS mutations; (ii) inability to complete MRI scans; (iii) Frontotemporal dementia (FTD)^19^; (iv) comorbidity of other neuropsychiatric disorders and (v) refusal to participate.

Finally, between April 2023 and February 2024, 53 ALS-ES patients were included. Moreover, 50 age- and sex-matched healthy controls (HCs) were recruited from the community and subjected to the same exclusion criteria as ALS patients.

### Clinical screening

Clinical screening was performed in ALS-ES patients. All patients’ demographic and clinical data were recorded, including age, sex, site of symptom onset, disease course, and upper motor neuron burden assessed using the Penn Upper Motor Neuron Score.^20^ The Amyotrophic Lateral Sclerosis Functional Rating Scale-Revised (ALSFRS-R) was used to assess disease severity.^21^ The information was further corroborated by an informant. Depression and anxiety were also assessed using the Hamilton Depression Rating Scale (HDRS) and Hamilton Anxiety Rating Scale (HARS), respectively.^21^

Moreover, clinical staging was evaluated using King's clinical staging system, and ALS patients were divided into the corresponding stages by a trained neurologist.^17^

Finally, longitudinal follow-up was performed in ALS patients. ALSFRS-R scores were assessed at follow-up, and FPR was defined as the slope of ALSFRS-R scores, calculated as the change between two visits divided by the disease duration between visits in months.

### Genetic testing

In the present study, all ALS patients underwent genetic testing.^21^ Similar to our previous studies, thirty-three ALS genes were screened via whole-exome sequencing.^21^

### MRI acquisition and voxel-based morphometry analysis

All MRI data were obtained on a 3.0 T magnetic resonance system (Prisma scanner, Siemens Medical Systems) with a 64-channel head coil. The process of MRI acquisition and voxel-based morphometry analysis is described in detail in the Supplementary material.

### Voxel-based QSM analyses

In the present study, the QSM reconstruction was performed using the QSMxT toolbox (https://qsmxt.github.io/QSMxT/), involving two steps: phase processing and susceptibility map estimation (workflow shown in Supplementary Fig. 1). Prior to processing, a brain mask was extracted from the QSM data using the BET function in FSL software (https://fsl.fmrib.ox.ac.uk/fsl). Subsequently, a rapid open-source minimum spanning tree (ROMEO) algorithm was used for phase unwrapping of multi-echo QSM data, followed by background field removal using the projection onto dipole field algorithm.^22,23^ Finally, dipole inversion for susceptibility map estimation was performed using a rapid two-step algorithm, which is compatible with mask-based QSM processing.^24^

After reconstruction, each subject's susceptibility map was normalized into Montreal Neurological Institute space using FLIRT & FNIRT functions in FSL software. Finally, all normalized susceptibility maps were smoothed with an 8-mm full width at half maximum Gaussian kernel to increase cortical sensitivity.

Finally, a region-of-interest (ROI) QSM analysis was performed. Similar to previous studies, based on the voxel-based whole-brain QSM analysis, significant regions between early-stage ALS patients and HCs were defined as ROIs.^5^ Then, the mean susceptibility value of each ROI was extracted and entered into partial correlation analyses with UMN score and FPR within the ALS group, with age, sex and TIV included as covariates.

### Statistical analysis

Continuous variables are reported as mean ± standard deviation, and categorical variables as frequency and proportion. Student's *t* test was used to compare continuous variables (with Mann–Whitney *U* test if necessary). Categorical variables were compared using chi-squared tests. A value of *P* < 0.05 indicated significance. Clinical data analysis was performed using SPSS version 20.0 (IBM Corp., Armonk, NY). Moreover, whole-brain QSM statistical analysis was conducted using a threshold-free cluster enhancement non-parametric permutation test with 5000 iterations, applying family-wise error (FWE) correction. A value of *P* < 0.05 indicated significance. This approach was implemented through the Randomise function in FSL. Then, partial correlation analysis was performed between clinical data and susceptibility values in ALS patients, and *P* < 0.05 was considered significant. For the above analyses, age, sex and TIV were used as covariates.

## Results

### Demographic and clinical information

In the present study, all included participants completed the clinical screening, cognitive assessment, genetic testing and MRI data acquisition. Three patients were found to carry pathogenic ALS-related mutations with an MAF < 0.5% in population databases, including two *SOD*1 mutation carriers and one FUS mutation carrier (Supplementary Table 1), and were excluded from the final analyses. Finally, 53 ALS-ES patients and 50 HCs were included in this study.

Moreover, in the present study, 51 ALS-ES patients completed the longitudinal follow-up, and the mean follow-up interval was 6.1 ± 1.3 months.

There were no significant differences in age or sex between the ALS-ES patients and HCs. Moreover, compared with HCs, HDRS and HARS scores were higher in ALS-ES patients. Demographic and clinical information of the early-stage ALS patients and HCs is shown in Table 1.

**Table 1:** Demographic and clinical features of early-stage ALS patients and HCs

	Patients with ALS (*n* = 53)	HCs (*n* = 50)	*P*-value
Age (years)	52.3 ± 11.6	52.4 ± 8.7	0.96
Men/Women (*n*)	31/22	29/21	0.96
Education (years)	9.7 ± 3.1	10.3 ± 2.9	0.32
ALS duration (month)	8.1 ± 2.7	-	-
Bulbar ALS onset *n*, (%)	15 (28.3)	-	-
ALSFRS-R score	43.2 ± 2.2	-	-
HARS	10.4 ± 7.8	3.4 ± 3.6	< 0.01
HDRS	6.9 ± 5.1	2.1 ± 2.3	< 0.01

### Whole-brain voxel-based QSM analysis and VBM analysis

**Figure 1:**
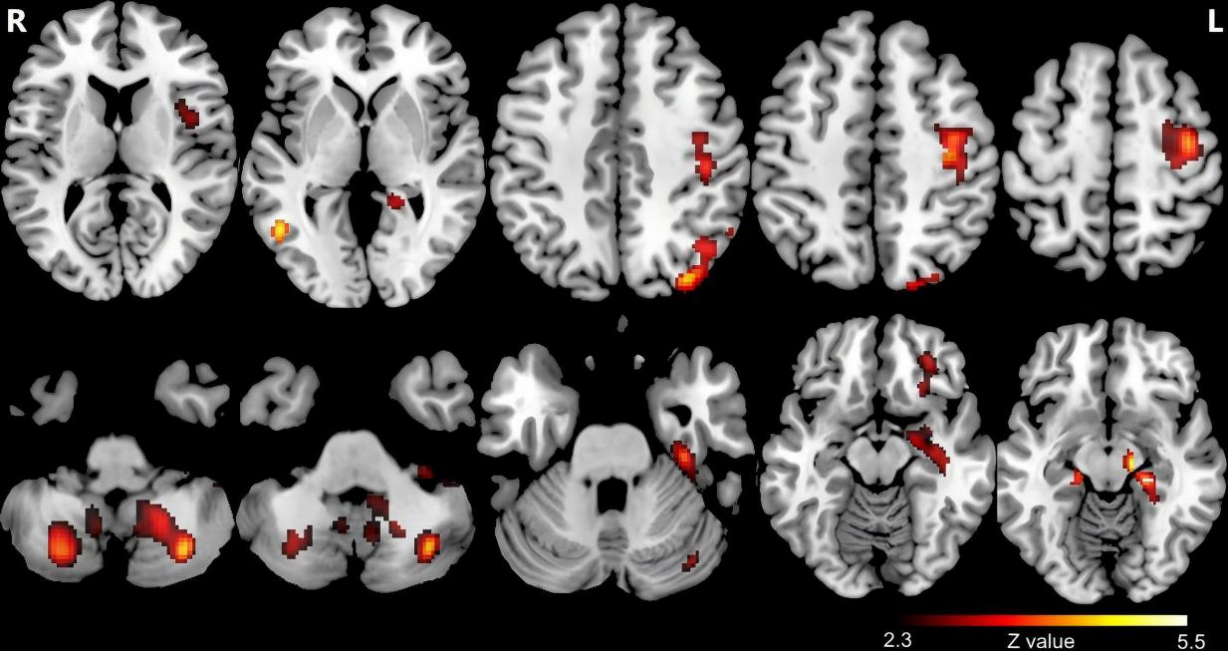
**Widespread brain iron accumulations in early-stage ALS patients.** For voxel-based whole-brain QSM analysis, compared with HCs, ALS-ES patients had significantly increased susceptibility values, mainly in the motor cortex, prefrontal cortex, hippocampus, amygdala, and cerebellar regions. Images are displayed in radiological convention (the left side of the image corresponds to the right hemisphere). The colour bar indicates Z values. R, right; L, left.

For whole-brain voxel-based QSM analysis, compared with HCs, ALS-ES patients had significantly increased susceptibility values in the right motor cortex regions, hippocampus, amygdala, cerebellum, prefrontal cortex, orbitofrontal cortex, insula, caudate, substantia nigra, red nucleus, postcentral cortex and left hippocampus after FWE correction (*P* < 0.05). The profiles of voxel-based whole-brain iron accumulation in early-stage ALS patients are presented in Fig. 1 and Supplementary Table 2.

Moreover, based on VBM analysis, GM alterations between early-stage ALS patients and HCs were not detected in our cohort.

### Correlation analyses

In the ALS-ES patients, the UMN score was significantly correlated with the mean susceptibility value of the right motor cortex (*r* = 0.504; *P* < 0.01). FPR was significantly correlated with the mean susceptibility value of the right motor cortex (r = 0.452, *P* < 0.01) and cerebellum (*r* = 0.429, *P* < 0.01) in early-stage ALS patients.

## Discussion

In a relatively large cohort, using voxel-based whole-brain QSM analysis, we first demonstrated a profile of early motor and extra-motor brain iron accumulation and their correlations with clinical disabilities in sporadic patients with early-stage ALS. We suggest that QSM is a promising neuroimaging approach for assessing early UMN damage and detecting early extramotor alterations in ALS. Moreover, motor cortex and cerebellar iron deposition were closely linked to disease progression in early-stage ALS. Thus, brain iron metabolism abnormalities may be promising therapeutic targets for slowing neurodegeneration in ALS.

To date, an increasing number of neuroimaging studies have used QSM to examine motor cortex iron abnormalities in ALS; however, these studies were commonly conducted in relatively advanced-stage patients.^5,7-11,25,26^ For example, Schweitzer *et al*. reported that motor cortex susceptibility was significantly increased in ALS patients compared with controls and suggested that QSM is a sensitive and specific quantitative biomarker of iron deposition in the motor cortex in ALS.^25^ In that study, the mean disease course was 29.3 months.^25^ Moreover, Bao *et al*., using SWI and 3D-T1-MPRAGE, reported significantly increased phase values in motor regions in ALS patients compared with HCs and suggested that iron accumulation in the motor region may improve diagnostic performance; however, the mean disease course was 20.19 months.^26^

Importantly, we further found that motor cortex iron accumulation can be detected in early-stage ALS; in particular, the mean disease course of our patients was 8.1 months. In our cohort, motor cortex susceptibility was closely linked to UMN score. Moreover, similar to previous studies, volumetric alterations assessed by VBM were not significantly different between ALS patients and HCs.^5,9^ Thus, our findings suggest that early motor cortex iron accumulation is an important feature of early-stage ALS. Collectively, these findings suggest that QSM is a promising neuroimaging approach that may provide valuable information for detecting early UMN damage and assisting early diagnosis *in vivo* in ALS.^5,7-11,25,26^ Further studies should verify our findings, particularly using QSM to assess motor cortex iron deposition in presymptomatic ALS.^18^

Moreover, we found that motor cortex and cerebellar iron accumulations were significantly related to faster FPR in early-stage ALS. To our knowledge, no prospective study has identified the contribution of brain iron deposition to disease progression in ALS.^5^ Only one retrospective SWI study reported that baseline progression rate was significantly related to susceptibility value in the motor cortex.^26^ However, that study did not assess extramotor iron alterations in ALS.^26^ Thus, it could not determine the relationship between extramotor iron deposition, such as in the cerebellum, and disease progression in ALS. Specifically, using structural MRI, Bede *et al*. recently reported that focal cerebellar degeneration is likely an important characteristic of ALS and suggested that pathognomonic symptoms, such as dysarthria, dysphagia, pseudobulbar affect and cognitive deficits, may be modulated, exacerbated or partially driven by cerebellar changes.^27^ Thus, these studies strongly support our findings.^26,27^ Collectively, improving motor cortex and cerebellar iron accumulation may be a promising treatment strategy for slowing disease progression in early-stage ALS.^26,27^

Importantly, as noted above, very few previous neuroimaging studies have used QSM to explore extra-motor brain iron changes in ALS.^5,9^ Recently, using QSM and structural MRI, Acosta-Cabronero *et al*. reported higher susceptibility in the motor cortex, left substantia nigra, right substantia nigra, globus pallidus and red nucleus in 28 ALS patients than in 39 HCs, while VBM did not reveal significant GM atrophy.^9^ Thus, they suggested that QSM is sensitive for detecting tissue alterations in ALS that may be relevant to pathology.^9^ Importantly, these findings provide initial evidence that not only motor cortex iron changes but also extra-motor brain iron abnormalities may occur in ALS.^9^ However, in that study, the mean disease course was 18 months; thus, whether extramotor iron deposition occurs in early-stage ALS remains unclear.^9^

Importantly, in this study, our findings suggest that early extra-motor brain iron deposition can be detected *in vivo* in early-stage ALS.^1,2,5,7,28^ Moreover, similar to previous studies, significant GM atrophy in motor or extramotor regions was not detected in our ALS patients.^5,9^ Specifically, the mean disease course of our patients was 8.1 months. Thus, our findings suggest that QSM is a useful approach for detecting early extra-motor alterations in early-stage ALS.^5^

Overall, our findings provide *in vivo* evidence that widespread brain iron deposition is an important characteristic of ALS. Thus, improving brain iron metabolism may be a promising therapeutic strategy for slowing neurodegeneration in ALS.^4-6^ In particular, iron chelation treatments were found to slow disease progression in ALS animals, further supporting our findings.^4-6^ Moreover, we suggest that QSM is a promising neuroimaging approach that may provide valuable information for assisting early diagnosis, assessing early extra-motor alterations and monitoring disease trajectories in ALS.^5^ However, further studies are still needed to confirm our findings.

Inevitably, the present study had several limitations. First, although the sample size was relatively large, this was a single-centre study.^5,7-11^ Thus, further multicentre or population-based studies are needed to verify our findings. Second, we only included sporadic ALS patients, and patients carrying known ALS-related mutations were excluded because of the sample size. Thus, we could not assess brain iron alterations in ALS patients with specific mutations, such as *SOD1* and *C9orf72*.^3,5^ Further studies should identify brain iron alterations in these patients. Third, although we observed widespread extra-motor susceptibility abnormalities, our correlation analyses were primarily focused on motor-related endpoints (UMN score and FPR) in line with the main objective of this brief report. Associations between extra-motor iron changes and non-motor symptoms (e.g. cognition, depression and anxiety) were not systematically evaluated as primary analyses; future studies with more comprehensive and standardized non-motor assessments are warranted.

In conclusion, we provide *in vivo* evidence that early motor and extra-motor brain iron accumulation are features of ALS and may play important roles in clinical disability. Thus, improving brain iron abnormalities may be a promising therapeutic strategy for slowing neurodegeneration in ALS. Moreover, our findings suggest that QSM may provide valuable information for identifying early UMN damage and monitoring disease trajectories in ALS. Further studies should verify our findings and extend to other neurodegenerative diseases in the future.

## Supplementary Material

fcag190_Supplementary_Data

## Data Availability

The anonymized data presented in this article are available at the request of a qualified investigator, after review by the corresponding author. Final approval will be granted by the Research Ethics Committee of the School of Medicine, Shandong University. No custom code was generated specifically for data analysis in this study. All analyses were conducted using established software packages and publicly available tools, as described in the Methods and Supplementary material.
