# Supplementary material for: Quantitative susceptibility mapping reveals widespread brain iron abnormalities in sporadic patients with early-stage amyotrophic lateral sclerosis
Source: Brain Commun. 2026 May 26;8(3):fcag190. doi: 10.1093/braincomms/fcag190 (PMC13256925; doi:10.1093/braincomms/fcag190)
Supplement: fcag190_Supplementary_Data [file fcag190_supplementary_data.docx]

**Supplementary Material**

**MRI acquisition**

All MRI data were obtained on a 3.0 T magnetic resonance system (Prisma scanner, Siemens Medical Systems) with a 64-channel head coil. Structural images of the whole brain were scanned using a three‑dimensional (3D) fast spoiled gradient-echo sequence: repetition time (TR) = 6.7 ms, echo time (TE) = 3.0 ms, matrix = 240×240×170, voxel size = 1mm×1mm×1mm, field of view (FOV) = 240 mm × 240 mm, and a total of 180 slices. FLAIR data were scanned using 2D acceleration, TR = 7000 ms, Flip Angle 90°, TE = 125 ms, acquisition matrix = 272 ×176, and slice thickness 6 mm. Moreover, the QSM sequence was performed with comprehensive brain coverage, employing parameters that included a TR of 50 ms and multiple echo times of 6.7, 10.6, 14.5, 18.4, and 22.4 ms, with a flip angle of 15°. The FOV was set to 240 × 240 mm, utilizing an acquired matrix of 384 × 269, resulting in a reconstructed in-plane resolution of 0.52 × 0.52 mm² and a slice thickness of 2.0 mm across 60 slices. The acquisition time for the QSM sequence was 5 minutes and 44 seconds. The GRAPPA technique was applied with a reduction factor of 2. These parameters, informed by prior literature, were selected to optimize the balance between signal-to-noise ratio (SNR), bandwidth, and spatial resolution while accommodating the complexities of brain imaging. All acquired images were visually inspected for motion artifacts by an experienced rater (Y.Y., with 10 years of experience in neuroimaging). Scans were excluded if they showed significant ghosting, blurring, or signal dropouts that could substantially affect QSM reconstruction or volumetric analysis.

**Voxel-based morphometry**
In the present study, voxel-based morphometry (VBM) analysis was also performed. The same CAT12-based workflow as in our previous work was used; however, key processing parameters are reported here. Briefly, VBM preprocessing and GM volume estimation were performed on T1-weighted images using the CAT12 (<http://dbm.neuro.uni-jena.de/cat12/>) toolbox implemented in SPM12 (Wellcome Centre for Human Neuroimaging, London, UK). Unless otherwise specified, default settings were used. VBM was performed to assess gray matter (GM) changes using the DARTEL approach, including bias-field correction and tissue segmentation, template creation based on all participants, spatial normalization to Montreal Neurological Institute (MNI) space, and modulation using the Jacobian determinants to preserve local volumetric information. The resulting GM maps were smoothed with an 8-mm full width at half maximum (FWHM) Gaussian kernel. ^1,2^

Quality control was performed by visual inspection for artifacts and segmentation errors and by checking CAT12’s internal quality metrics (image quality rating and sample homogeneity). No participant was excluded based on VBM quality control.

Voxel-wise group comparisons were conducted using a general linear model, with age, sex, and total intracranial volume (TIV) included as covariates. Statistical significance was assessed across the whole brain at the voxel level using family-wise error (FWE) correction (p < 0.05).

Moreover, using structural MRI data, total intracranial volume (TIV) was calculated for each subject for further analysis. TIV was estimated in CAT12 as the sum of GM, white matter, and cerebrospinal fluid volumes derived from tissue segmentation, and was used as a covariate in the VBM analysis.

**Participant recruitment**Between April 2023 and February 2024, 59 ALS patients were assessed for eligibility. Of these, three were excluded due to concomitant frontotemporal dementia (FTD), and three additional patients were excluded because pathogenic ALS-related mutations were identified on genetic testing (two SOD1, one *FUS*). Therefore, 53 newly diagnosed early-stage sporadic ALS patients were included in the baseline imaging analyses. Fifty age- and sex-matched healthy controls were recruited. Longitudinal follow-up was completed in 51 of the 53 ALS-ES patients.

**Genetic testing**
In this study, whole exome sequencing (WES) was performed on all ALS patients to screen for ALS-associated genes. The 33 ALS-related genes that were tested are as follows:
*SOD1, ALS2, SETX, FUS, VAPB, TARDBP, OPTN, VCP, UBQLN2, SIGMAR1, FIG4, CHMP2B, PFN1, NEFH, PRPH, TFG, TAF15, GRN, CHCHD10, TUBA4A, TBK1, NEK1, GLE1, MATR3, CCNF, ANXA11, HNRNPA1, SQSTM1, ERBB4, TIA1, SPG11, ANG,* and *KIF5A*.

**Comparison of age at onset across regions/continents**
In the present cohort, the mean age at onset was 52.3 ± 11.6 years. This onset-age profile is comparable to that reported in a large mainland Chinese ALS cohort.^3^ In contrast, studies from Western countries with predominantly Caucasian populations generally report a higher age at onset, often in the low-to-mid 60s, as summarized in the literature.

**Baseline ALSFRS-R in early-stage/King stage cohorts**
Our cohort consisted of newly diagnosed, early-stage sporadic ALS patients (King’s stage 1) meeting the Gold Coast criteria. At baseline, the ALSFRS-R score was 43.2 ± 2.2 and disease duration was 8.1 ± 2.7 months, reflecting relatively preserved function. Prior early-stage/King stage–based studies reported comparable baseline ALSFRS-R values, including 43.6 ± 1.6 in early-stage (King’s stage 1) ALS (Liu et al., *Brain*, 2024)^4^ and 45 (IQR 43–46) in King’s stage 1 ALS (Canosa et al., *Eur J Nucl Med Mol Imaging*, 2021).^5^

**References**

1. Wang Y, Shen D, Hou B, Sun X, Yang X, Gao J, Liu M, Feng F, Cui L. Brain structural and perfusion changes in amyotrophic lateral sclerosis-frontotemporal dementia patients with cognitive and motor onset: a preliminary study. Brain Imaging Behav. 2022 Oct;16(5):2164-2174.

2. Shen D, Hou B, Xu Y, et al. Brain Structural and Perfusion Signature of Amyotrophic Lateral Sclerosis With Varying Levels of Cognitive Deficit. Front Neurol. 2018 May 24;9:364.

3. Chen YP, Yu SH, Wei QQ, et al. Role of genetics in amyotrophic lateral sclerosis: a large cohort study in Chinese mainland population. J Med Genet. 2022 Sep;59(9):840-849.

4. Liu S, Sun X, Ren Q, et al. Glymphatic dysfunction in patients with early-stage amyotrophic lateral sclerosis. Brain. 2024 Jan 4;147(1):100-108.

5. Canosa A, Calvo A, Moglia C, et al. Brain metabolic changes across King's stages in amyotrophic lateral sclerosis: a 18F-2-fluoro-2-deoxy-D-glucose-positron emission tomography study. Eur J Nucl Med Mol Imaging. 2021 Apr;48(4):1124-1133.

Supplementary Figure 1. Schematic overview of the quantitative susceptibility mapping (QSM) processing pipeline.


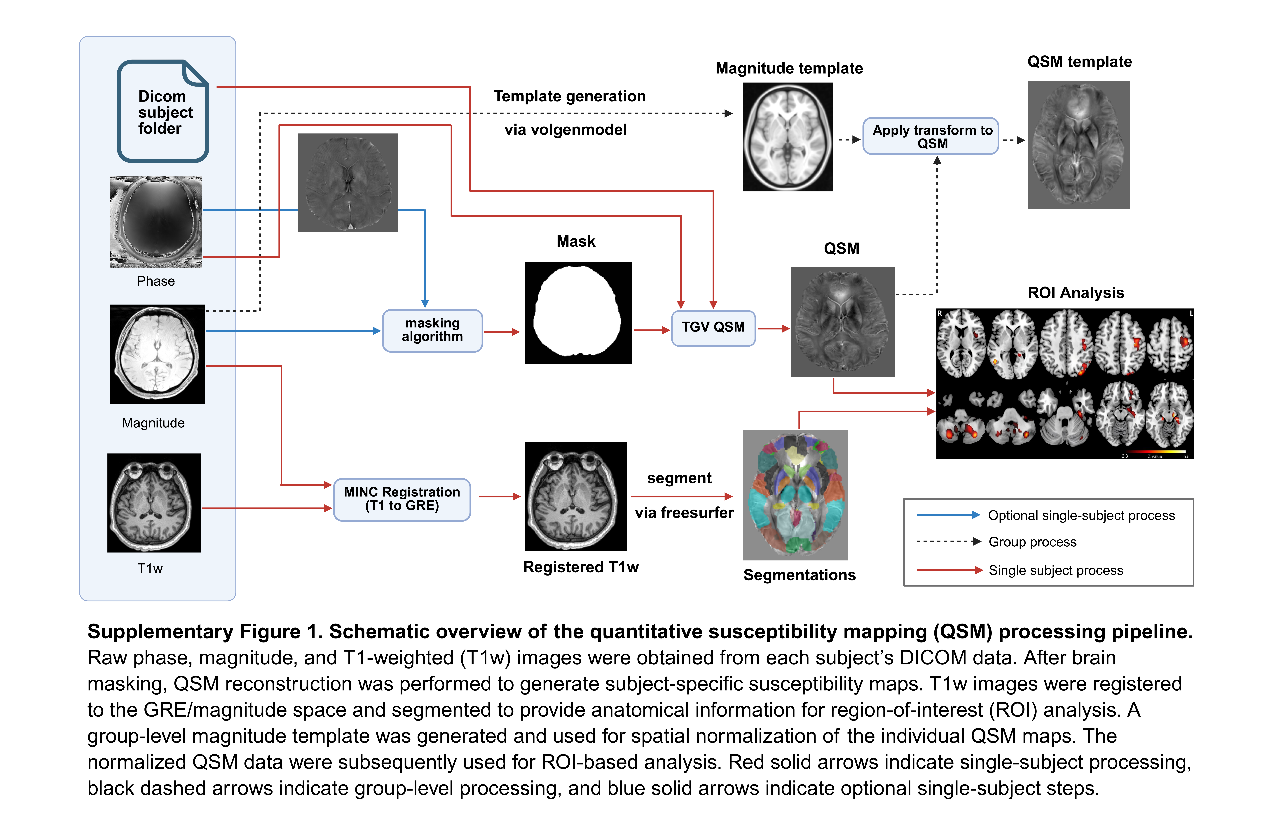


Supplementary Table 1. Genetic data of patients carrying ALS gene mutations

| No. | Mutant gene | Nucleotide change | Amino acid change |
| --- | --- | --- | --- |
| Patient 1 | *SOD1* | c.404G>C | p.S135T |
| Patient 2 | *SOD1* | c.143T>C | p.V48A |
| Patient 3 | *FUS* | c.1574C>T | p.P525L |

Abbreviations: ALS = amyotrophic lateral sclerosis.

Supplementary Table 2. Results of voxel-wise whole-brain QSM analysis

| **Cluster** | **Regions (Size)** | **Peak Coordinate** | | | **Z Value** | **P Value** |
| --- | --- | --- | --- | --- | --- | --- |
|  |  | **X** | **Y** | **Z** |  |  |
| 01 | Cerebellum (2396) | 32 | -68 | -44 | 4.5524 | 0.0024 |
| 02 | Hippocampus R (316)  Para-Hippocampal R (81)  Amygdala R (132) | 34 | -24 | -30 | 4.0562 | 0.0046 |
| 03 | Temporal R (45) | 50 | 24 | -22 | 2.8026 | 0.0068 |
| 04 | Prefrontal R (205)  OFC (236)  Insula R (33) | 30 | 42 | -14 | 3.3037 | 0.0064 |
| 05 | Precentral R (125) | 56 | 18 | 34 | 5.1114 | 0.0046 |
| 06 | Caudate R (24) | 18 | -2 | 16 | 2.5641 | 0.0068 |
| 07 | Hippocampus R (66)  Para-Hippocampal R (127) | 30 | -40 | -20 | 4.2049 | 0.0070 |
| 08 | Hippocampus L (23)  Para-Hippocampal L (22) | -18 | -24 | -12 | 3.7649 | 0.0086 |
| 09 | SN R (31)  RN R (19) | 14 | -18 | -10 | 5.3195 | 0.0062 |
| 10 | Temporal Mid L (169) | -54 | -56 | 8 | 4.7717 | 0.0084 |
| 11 | Parietal (223) | 34 | -78 | 42 | 4.6385 | 0.0082 |
| 12 | Precentral R (481)  Prefrontal (135) | 36 | -18 | 54 | 4.5128 | 0.0062 |

Abbreviations: QSM = quantitative susceptibility mapping; R = right; L = left; OFC = orbitofrontal cortex; SN = substantia nigra; RN = red nucleus.
